# Effect of annealing temperature on wettability of TiO_2_ nanotube array films

**DOI:** 10.1186/1556-276X-9-621

**Published:** 2014-11-18

**Authors:** Lei Yang, Miao Zhang, Shiwei Shi, Jianguo Lv, Xueping Song, Gang He, Zhaoqi Sun

**Affiliations:** 1 School of Physics and Materials Science, Anhui University, Hefei 230601, People's Republic of China; 2 New Star Institute of Applied Technology, Hefei 230031, People's Republic of China; 3 Department of Physics and Electronic Engineering, Hefei Normal University, Hefei 230061, People's Republic of China

**Keywords:** TiO_2_ nanotube arrays, Annealing temperature, Wettability, Photoinduced hydrophilicity

## Abstract

Highly ordered TiO_2_ nanotube array (TN) films were prepared by anodization of titanium foil in a mixed electrolyte solution of glycerin and NH_4_F and then annealed at 200°C, 400°C, 600°C, and 800°C, respectively. The samples were characterized by X-ray diffraction (XRD), scanning electron microscopy (SEM), water contact angle (WCA), and photoluminescence (PL). It was found that low temperature (below 600°C) has no significant influence on surface morphology, but the diameter of the nanotube increases from 40 to 50 nm with increasing temperature. At 800°C, the nanotube arrays are completely destroyed and only dense rutile film is observed. Samples unannealed and annealed at 200°C are amorphous. At 400°C, anatase phase appears. At 600°C, rutile phase appears. At 800°C, anatase phase changes into rutile phase completely. The wettability of the TN films shows that the WCAs for all samples freshly annealed at different temperatures are about 0°. After the annealed samples have been stored in air for 1 month, the WCAs increase to 130°, 133°, 135°, 141°, and 77°, respectively. Upon ultraviolet (UV) irradiation, they exhibit a significant transition from hydrophobicity to hydrophilicity. Especially, samples unannealed and annealed at 400°C show high photoinduced hydrophilicity.

## Background

In 1997, Wang et al. [1] reported that ultraviolet (UV) illumination of TiO_2_ surfaces could produce a highly hydrophilic surface which was named as super-hydrophilicity. Since then, hydrophilic TiO_2_ materials have attracted attention for many practical applications such as self-cleaning and antifogging materials [2]. It is well known that the wettability of TiO_2_ thin films strongly depends on the preparation methods and annealing conditions, which have a decisive influence on the physical and chemical properties of TiO_2_ thin films [3,4]. Therefore, it is necessary to investigate the effects of the preparation process and annealing conditions on the wettability of the films.

TiO_2_ thin films can be prepared by techniques such as sol-gel method [5], template method [6], sputtering method [7,8], vapor and liquid phase deposition [9], and electrochemical deposition [10,11]. In 1999, Zwilling and co-workers grew the first highly ordered TiO_2_ nanotube array (TN) films by anodization of titanium foil in a HF-containing electrolyte, which has attracted wide interest [12]. Thermal treatment of TiO_2_ and TiO_2_ nanotubes provides a facile route to control grain size, particle morphology, microstructures, phase composition, and surface photoelectrochemical properties via adjusting experimental parameters such as temperature, time, and atmospheres [13-17]. For the wettability of TN films, previous works mainly focus on tubular geometry modified with low surface energy materials. In this work, by changing the annealing temperature, the effects of phase transition combined with nanotubular morphology on the wettability of TN films were investigated and discussed.

## Methods

### Preparation of samples

Titanium foils (0.2 mm thick, 99.6% purity), glycerin (analytical reagent (A.R.), 99.0%), and NH_4_F (A.R., 96.0%) were used in this experiment. Titanium foils were degreased by sonicating in acetone and deionized (DI) water, followed by rinsing with DI water and drying in air. Anodization of titanium foils was carried out at room temperature using a two-electrode system (2-cm separation) with a direct current power supply. The samples were anodized in solutions containing 0.175 M NH_4_F consisting of mixtures of DI water and glycerol (volume ratio 1:20) at 30 V for 3 h, similar to the method described by Schmuki and co-worker [18]. After fabrication, the samples were rinsed in DI water and dried in air. Thermal annealing was performed in ambient air for 2 h at 200°C, 400°C, 600°C, and 800°C, respectively.

### Characterization of samples

A field emission scanning electron microscope (FE-SEM; Hitachi S4800, Hitachi, Ltd., Chiyoda, Tokyo, Japan) was employed for the morphological characterization of the TN films. The crystal structure of the TN films was checked by means of X-ray diffraction (XRD; MACM18XHF, MAC Science, Yokohama, Japan). The water contact angle (WCA) of the films was measured by a homemade WCA apparatus, which was performed in ambient conditions (293 K, relative humidity 60%). The volume of the water droplets used for the WCA measurements was 3 μL. Each WCA measurement was repeated four times at different places on the sample. To study the photoinduced hydrophilicity, samples were irradiated using a UV mercury lamp (*λ* =365 nm) with an intensity of 1.0 μW/cm^2^. After each irradiation time interval, a 3-μL water droplet was placed on the irradiated area and the corresponding angle was measured. The WCA can be calculated from Equation 1 [19]:

(1) $$\theta =\text{arctan}\frac{4HL}{L^{2}-4H^{2}}$$

where *L* and *H* are the diameter and height, respectively, of the spherical crown of the droplet dropped on the surface of the thin films. The experimental error of the measurements is ±1°.

## Results and discussion

### Microstructure and morphology

**Figure 1:**
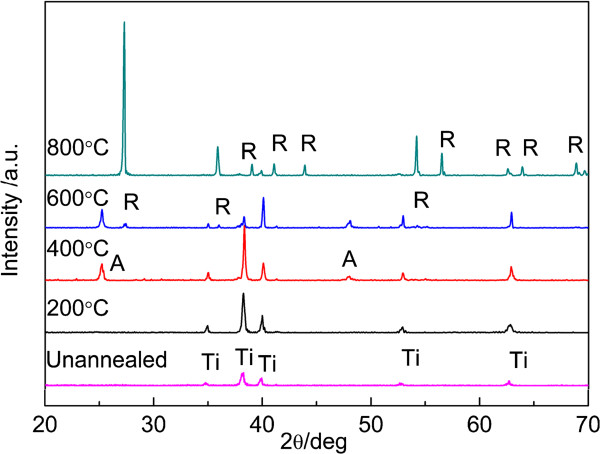
XRD patterns of TN films unannealed and annealed at 200°C, 400°C, 600°C, and 800°C.

Figure 1 shows the XRD patterns of the TN films annealed at various temperatures in air. It is obvious that only the diffraction peaks of the titanium substrate can be found when the annealing temperature is 200°C (JCPDS No. 44-1294); it is suggested that these TNs are amorphous. For the sample annealed at 400°C, two obvious peaks at 2*θ* =25.3° and 48.1° corresponding to (101) and (200) plane diffraction of anatase TiO_2_ (JCPDS No. 21-1272) have been clearly observed except the diffraction peaks of the titanium substrate. For the sample annealed at 600°C, small peaks at 2*θ* =27.4°, 36.1°, and 54.3° corresponding to (110), (101), and (201) plane diffraction of rutile (JCPDS No. 21-1276) have been observed. For the sample annealed at 800°C, anatase phase changes into rutile phase completely. Further observation indicates that the diffraction peaks of titanium almost vanish at 800°C. A similar phenomenon was also reported by Grimes et al., and they suggested that titanium is directly oxidized and transformed into rutile titania at high temperature [20].

**Figure 2:**
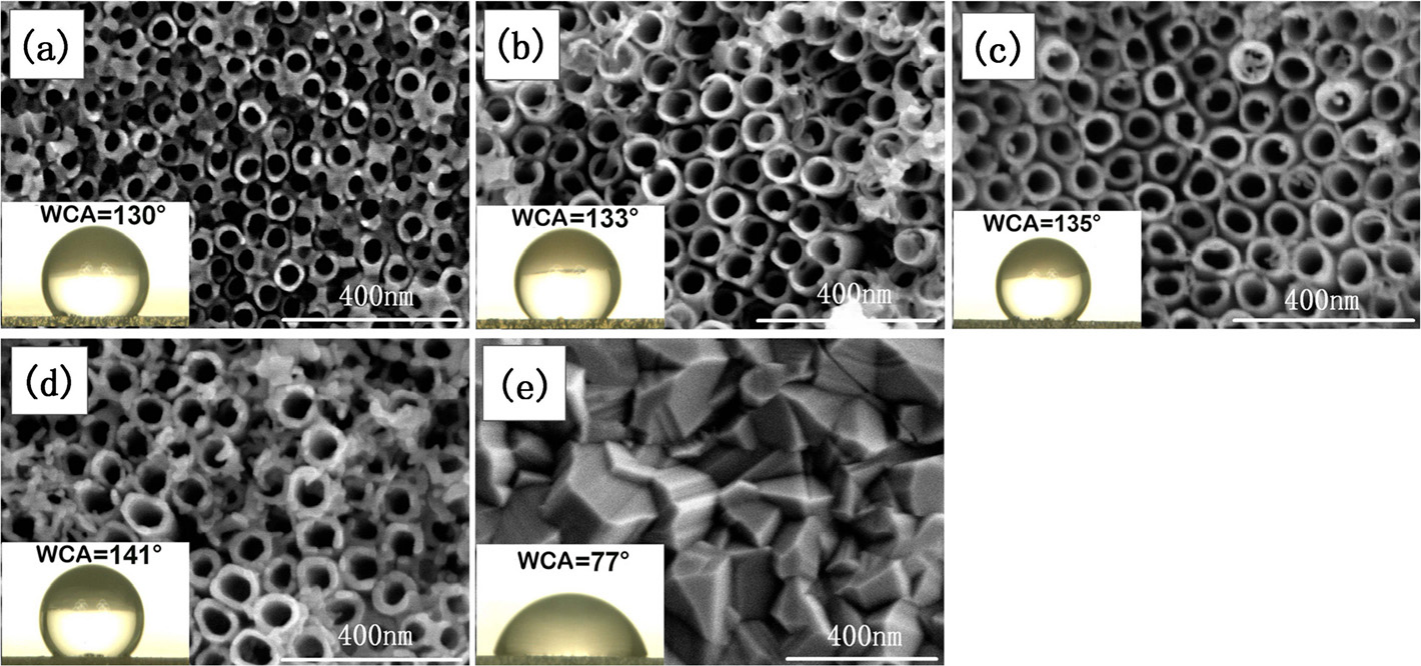
**SEM images of TN films (a) unannealed and annealed at (b) 200°C, (c) 400°C, (d) 600°C, and (e) 800°C.** The insets are optical images of water droplets on the sample surface, during the WCA measurement.

Figure 2 presents the scanning electron microscopy (SEM) images of TN films (a) unannealed and annealed at (b) 200°C, (c) 400°C, (d) 600°C, and (e) 800°C for 2 h. As shown in Figure 2, all samples exhibit highly ordered TNs except the sample annealed at 800°C. It should be noted that the unannealed TNs have an average diameter of 40 nm. When the samples were annealed at 200°C and 400°C, the TNs' surface morphology remains uniform compared to that of the original TNs; however, the average diameter of the nanotubes increases to 50 nm. This may be related to the dehydration reaction that occurs when the phase transforms from amorphous to anatase [21]. At 600°C, the surface morphology of the TN sample has an obvious change, which is ascribed to the nucleation of rutile phase. When the annealing temperature grows up to 800°C, the TNs are completely destroyed and dense rutile film is observed (Figure 2e), which is attributed to the fact that high temperature causes the growth of rutile crystallites.

### Wettability

To characterize the wettability, both WCA and sliding angle (SA) are necessary. However, in our experiment, the films can hold water droplets through adhesive forces, even when the layer is tilted vertically or turned upside down; thus, surface wettability is evaluated by WCA measurement. Herein, the WCAs for all samples freshly annealed at different temperatures are about 0°. That is, the water droplet entirely spreads. This finding implies that the intrinsic state of a titania film is hydrophilic [22,23] and this is in line with the classical Wenzel model [24]. In this model, it is assumed that a droplet fills up a rough surface, therefore, forming a fully wetted contact, which depends on the roughness factor and the surface free energy. In other words, the Wenzel model represents a classical situation of a droplet on a ‘flat’ surface just modified by a roughness factor. Herein, when a high surface energy material combines with micro- and nanoscale roughness, a super-hydrophilic surface is obtained.

After the samples annealed at different temperatures have been stored in air for 1 month, the WCAs increase to 130°, 133°, 135°, 141°, and 77°, respectively (insets in Figure 2). This wetting behavior may be ascribed to the replacement of the chemisorbed hydroxyl groups with the oxygens [25] and the adsorption of organic contaminants on the TN film surface in air ambience [26]. More importantly, the WCA of TN films is larger than the previous results achieved in TiO_2_ surface structures [22,25,27]. This behavior may be attributed to the ordered nanotubular structure and the great surface area. And this situation is described by the Cassie and Baxter model [28]. For a low surface energy material, Cassie and Baxter assumed that the liquid does not completely permeate the roughened solid. As a result, air pockets are trapped inside the features underneath the liquid, which sits above a composite surface made of solid and air. In the Cassie and Baxter model, the contact angle (*θ*^∗^) can be expressed as:

(2) $$\text{cos}\theta^{*}=\left(1-f_{v}\right)\left(1+\text{cos}\theta \right)-1$$

where *f*_v_ is the area fraction of vapor on the surface and *θ* is the contact angle measured on the flat surface. As *f*_v_ is always lower than unity, this model always predicts enhancement of hydrophobicity only [29].

As shown in Figure 2a,b,c, the average pore size and the space among the tubes increase with increasing annealing temperature up to 400°C. Meanwhile, the WCA increases from 130° to 135°. This can be explained naturally by the Cassie and Baxter model, that the trapped air among tubes would increase the liquid-air contact area, thereby forming bigger WCA. From Figure 2d, at 600°C, the WCA is up to 141° and the sample surface roughness may be enhanced because of the breaking of nanotubes. Therefore, the increase of WCA may be attributed to the increase of area fraction of vapor on the surface *f*_v_ and the enhanced roughness. When the sample was annealed at 800°C, the WCA decreases to 77°, caused by collapsing of the nanotubular structure.

### Photoinduced wettability

**Figure 3:**
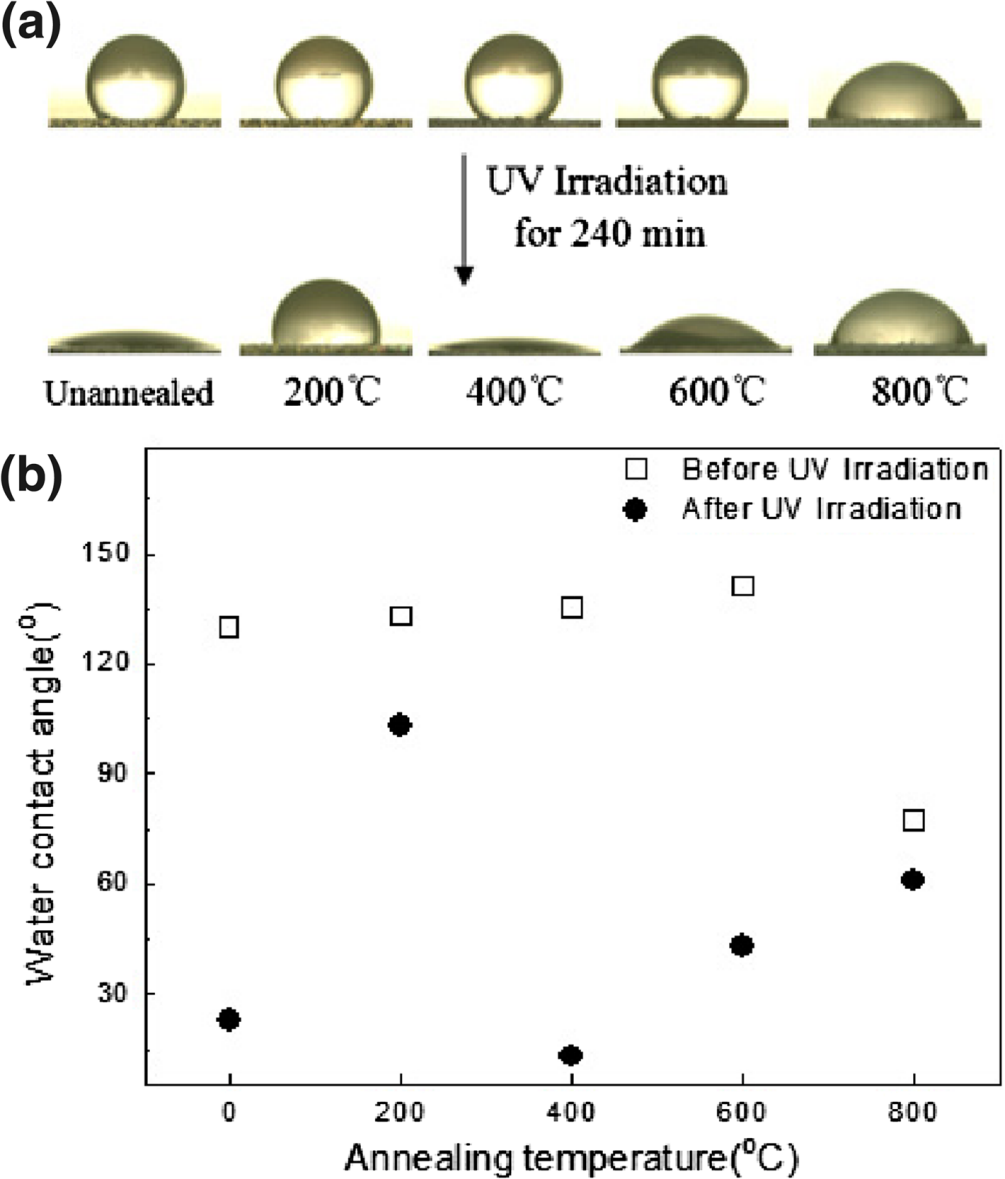
**Photographs of water droplet shape and the corresponding WCA.** Photographs of water droplet shape on the TN films before and after UV irradiation **(a)**. The corresponding WCA were plotted in **(b)**.

UV irradiation, as an effective surface treatment method, has been effectively used to create hydrophilic surfaces [1,30]. Table 1 and Figure 3 show the changes in the WCA under UV irradiation. The TN films annealed at different temperatures in air show WCAs of 23°, 103°, 13°, 43°, and 61°, respectively, after UV irradiation for 240 min in ambient conditions. It is found that the WCA decreases as the annealing temperature grows from 200°C to 400°C and increases as the temperature grows above 400°C. For the unannealed TN films, the WCA is bigger than that for the films annealed at 400°C and smaller than that for the films annealed at 600°C. Therefore, the optimal annealing temperature for photoinduced hydrophilicity is around 400°C. As is mentioned above, the crystal structure and the surface morphology are different for the samples annealed at different temperatures. Herein, the change in the WCA can be explained in terms of two factors: On the one hand, the wettability is dependent on the film's crystal structure. When the sample was annealed at 200°C, the microstructure of TNs is amorphous, which shows poor photoinduced hydrophilicity. When the sample was annealed at 400°C, anatase phase appears, resulting in high photoinduced hydrophilicity. However, when the sample was annealed at 600°C, the anatase TiO_2_ starts to transform gradually to rutile TiO_2_, leading to the rise in the WCA. This is because hydrophilicity is related to the density of surface hydroxyl. The surface hydroxyl can easily combine with water molecule to form hydrogen bond, resulting in hydrophilicity. So, the more surface hydroxyl, the better hydrophilicity. Under the same UV illumination, although changes in hydrophilicity have been observed on both the anatase and rutile TiO_2_ surface [31], the former with more surface hydroxyl is more active in achieving hydrophilic features than the latter with less surface hydroxyl. On the other hand, the wettability is also dependent on the film's surface morphology. When the sample was annealed at 800°C, the nanotubular structure collapses, causing the least WCA reduction rate.

**Table 1:** WCA reduction rate of the samples annealed at different temperatures

		**Annealing temperature (°C)**			
	**Unannealed**	**200**	**400**	**600**	**800**
Before UV irradiation WCA (^o^)	130	133	135	141	77
After UV irradiation WCA (^o^)	23	103	13	43	61
Contact angle reduction rate (%)	82.0	22.4	90.4	69.3	20.4

Furthermore, from Table 1 and Figure 3, it is noteworthy that the unannealed sample possesses a WCA of 23° after UV irradiation, which is much smaller than that annealed at 200°C. Combining XRD analysis with the SEM image, it can be seen that they are both amorphous and almost have the same surface morphology. Sun et al. proposed a mechanism of the photoinduced hydrophilicity of TiO_2_, which originates from an increase of the hydroxyl groups on the TiO_2_ surface. Some of the photogenerated holes diffuse to the surface of TiO_2_ and react with lattice oxygen, leading to the formation of surface oxygen vacancies. Water molecules are feasible to occupy those oxygen vacancies to produce surface-adsorbed hydroxyl groups, which tend to make the surface hydrophilic [22]. Therefore, variation of the WCA should be primarily attributed to the difference in their surface defective sites.

**Figure 4:**
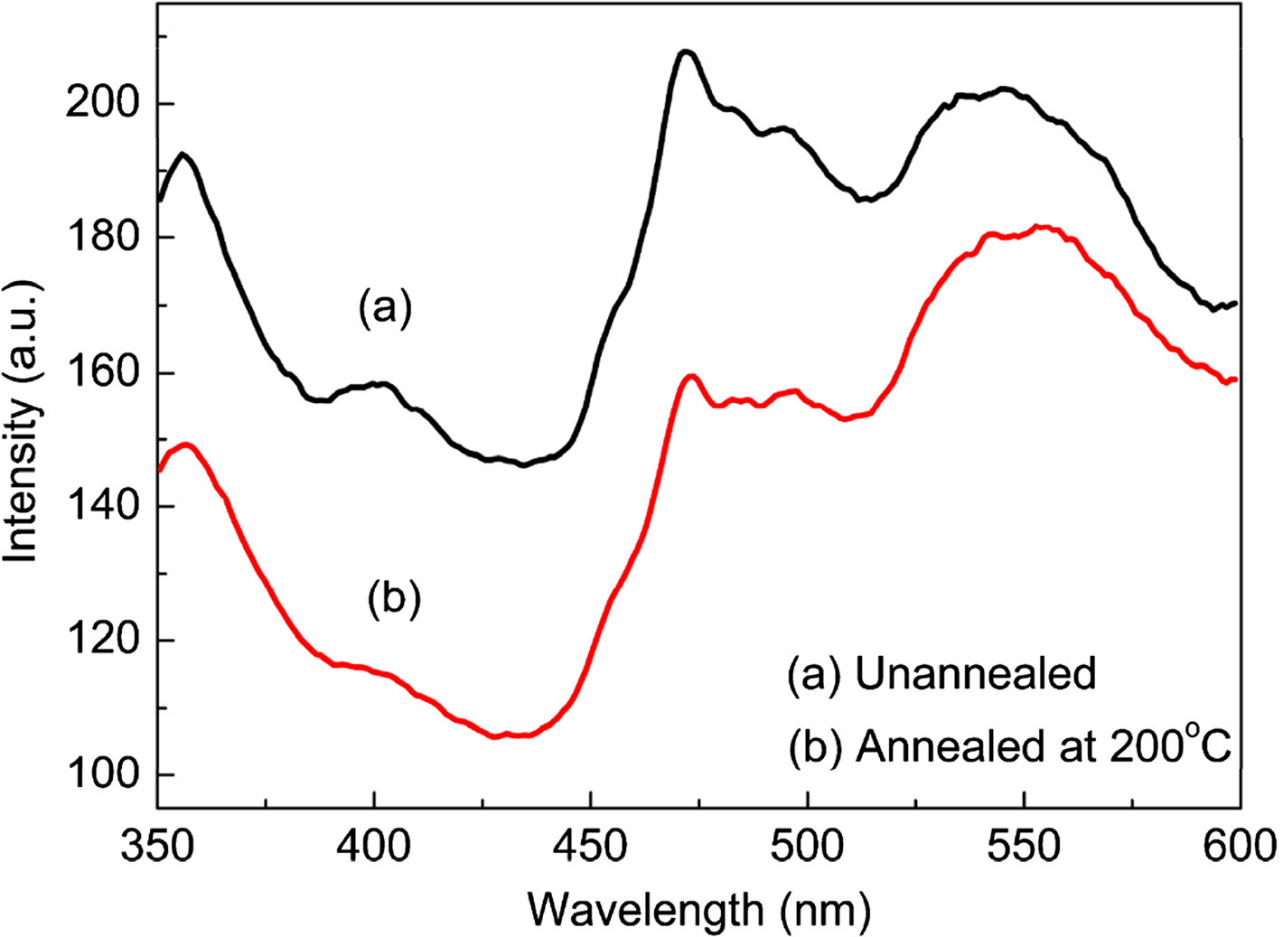
**Photoluminescence spectra of TN films (a) unannealed and (b) annealed at 200°C after UV irradiation for 60 min.**

As photoluminescence (PL) properties of the materials closely correspond to the surface features of the material that could be changed remarkably by the annealing processes [32], we measured the PL spectra of TN films upon UV irradiation for 60 min. The PL spectra of TN films were obtained by using an excitation wavelength of 325 nm in the range from 350 to 600 nm at room temperature. As shown in Figure 4, the unannealed TN films display two visible luminescence bands located at 470 and 540 nm which decrease in intensity in the sample annealed at 200°C. It has been reported that the peaks located in the visible luminescence band are correlated to oxygen vacancies on the surface of TiO_2_ and that the intensity of the emission peaks increases with the defect levels [33-35]. So, we can surmise that the oxygen vacancy defects decrease in TN films with annealing temperature growth up to 200°C. Therefore, the photoinduced hydrophilicity of the TN films annealed at 200°C weakened.

## Conclusions

In summary, TN films have been prepared by anodization on a pure titanium foil. The crystal structure of TN films transforms from amorphous to anatase and rutile with annealing temperature increasing from room temperature to 800°C. The study of the wettability of the films shows that the WCAs for all samples freshly annealed at different temperatures are about 0°. After the annealed samples have been stored in air for 1 month, the WCA has a significant increase. Upon UV irradiation, it exhibits a significant transition from hydrophobicity to hydrophilicity. The study of the wettability of the films shows that the wettability of the TN films relates to the superficial cleanliness, crystal structure, oxygen vacancy defects, and surface morphology.

## Competing interests

The authors declare that they have no competing interests.

## Authors’ contributions

ZS and GH designed the experiments. LY, MZ, and SS carried out the experiments. LY wrote the paper. XS and JL analyzed the results and participated in the revision of the manuscript. ZS and JL proofread the manuscript and corrected the English. All authors read and approved the final manuscript.
